# A New Perspective on Cooking Stove Loss Coefficient Assessment by Means of the Second Law Analysis

**DOI:** 10.3390/e24081019

**Published:** 2022-07-23

**Authors:** Lomena Mulenda Augustin, Sumuna Temo Vertomene, Ndaye Nkanka Bernard, Amsini Sadiki, Mbuyi Katshiatshia Haddy

**Affiliations:** 1 Centre de Recherche en Energies Renouvelables, Faculté Polytechnique, Université de Kinshasa, Avenue de l’Université N° 01, Commune de Lemba, BP 127 Kinshasa, Democratic Republic of the Congo or augustinlomena@gmail.com (L.M.A.); temo.sumuna@unikin.ac.cd (S.T.V.); haddy.mbuyi@unikin.ac.cd (M.K.H.); 2 Centre d’Etudes et de Recherches sur les Energies Renouvelables Kitsisa-Khonde (CERERK), ISTA-Kinshasa, Avenue Aérodrome N° 3930, Commune de Barumbu, BP 6593 Kinshasa, Democratic Republic of the Congo; ndaye.nkanka@ista.ac.cd; 3 Institute for Energy and Power Plant Technology, Technische Universität Darmstadt, 64287 Darmstadt, Germany; 4 Laboratoire de Modélisation Mécanique, Energétique et Matériaux, ISTA-Kinshasa, Avenue Aérodrome N° 3930, Commune de Barumbu, BP 6593 Kinshasa, Democratic Republic of the Congo; 5 Institute for Reactive Flows and Diagnostics (RSM), Technische Universität Darmstadt, 64287 Darmstadt, Germany

**Keywords:** woodburning cooking stove, fuel-burning rate, buoyancy, loss coefficient, entropy-generation rate, Carnot factor, exergy

## Abstract

The chimney effect taking place in biomass cooking stoves results from a conversion process between thermal and mechanical energy. The efficiency of this conversion is assessed with the stove loss coefficient. The derivation of this quantity in cooking stove modelling is still uncertain. Following fluid mechanics, this loss coefficient refers to an overall pressure drop through stove geometry by performing an energy balance according to the first law of thermodynamics. From this approach, heat-transfer processes are quite ignored yet they are important sources of irreversibilities. The present work takes a fresh look at stove loss coefficient assessment relying on the second law of thermodynamics. The purpose in this paper is to identify the influence of operating firepower level on flow dynamics in biomass natural convection-driven cooking stoves. To achieve that, a simplified analytical model of the entropy-generation rate in the flow field is developed. To validate the model, experiments are conducted first on a woodburning stove without cooking pot to better isolate physical processes governing the intrinsic behaviour of the stove. Then, for the practical case of a stove operating with a cooking pot in place, data from published literature have served for validation. In particular, mass-flow rate and flue gas temperature at different firepower levels have been monitored. It turns out that losses due to viscous dissipations are negligible compared to the global process dissipation. Exergy analysis reveals that the loss coefficient should rather be regarded from now as the availability to generate flow work primarily associated with the heat-transfer Carnot factor. In addition, the energy flux applied as flow work has to be considered as pure exergy that is lost through consecutive energy-transfer components comprising the convective heat transfer to the cooking pot. Finally, this paper reports a satisfactory agreement that emerged between the exergy Carnot factor and the experimental loss coefficient at different fuel-burning rates.

## 1. Introduction

The combustion-induced driven flow is the phenomenon that occurs in traditional cooking stoves widely used in the developing world, and mainly in rural areas, where the major portion of the population uses biomass fuel as the primary source of energy [1,2,3]. Inefficient stoves are important sources of emissions of pollutants hazardous to human health, such as carbon monoxide (CO), particulate matters (PM) and polycyclic aromatic hydrocarbons (PAHs) [4,5,6,7]. The Clean Cooking Alliance reports that every year four million people die from illnesses associated with smoke from cooking activities and, at the same time, burning woodfuels contributes to about 2% of global CO${}_{2}$ emissions [8]. Thus, the challenge for clean cooking designers is to create user-friendly appliances that can maintain high overall efficiency and reduce harmful emissions to levels low enough to ensure health, environment and climate co-benefits [7].

Given its importance, the conception of clean cooking stoves is now increasingly deserving of the attention of researchers [9,10,11]. Starting in the 1980s, early modelling efforts have been initiated to design more efficient cooking stoves. Since then, two types of models have emerged from researchers. The primary type is a zonal model in which conservation of mass, momentum and energy are applied to different zones within the stove [9]. In the second type of model, Computational Fluid Dynamics (CFD) is used to represent detailed informations inside the computational domain, such as regions of high soot and CO concentrations [12,13,14]. While remarkable progress has been made in the modelling of cooking stoves, many questions remain unanswered.

**Figure 1:**
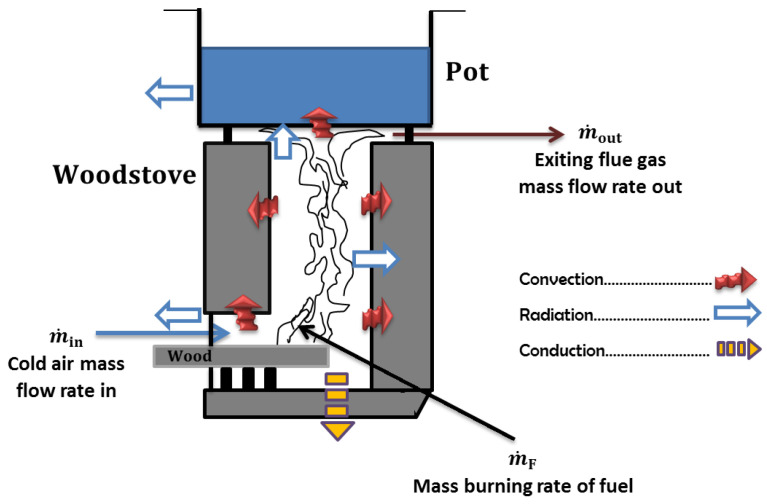
Schematic of a chimney woodstove cross-section with different heat-transfer modes [15].

Figure 1 presents significant features of a natural draft stove burning wood fuel including a horizontal combustion chamber, and under the cooking pot there is an insulated short chimney, inside which takes place a buoyant flow of hot gases [11]. Buoyancy occurring in the stove results from a conversion process between two forms of energy (internal and mechanical) conserving the overall energy according to the first law of thermodynamics. The efficiency of this conversion is globally assessed by the stove loss coefficient. The derivation of this quantity in cooking stove modelling is still uncertain. How this stove flow loss coefficient varies when fire takes place under different woodburning rates remains a challenging concern.

To better understand the importance of the issue, it is worth mentioning in this study that in woodstove modelling, great attention has always been focused on the influence of design parameters such as geometry or insulation materials [16,17,18,19], whereas little prior consideration has been devoted to the operating firepower level impacting aerodynamics and chemistry in the stove. The firepower–performance dependency becomes a key issue whose interest has been growing only in the last 10 years [20]. The works of Agenbroad et al. [21,22,23] represent an important benchmark in identifying the influence of operating firepower on woodburning stove behaviour. Moving beyond empirical/observational approaches, these authors developed and validated experimentally on steady state assumption an analytical and simplified stove flow theory that predicts mass-flow rate and exhaust-gas temperature from stove design and operating firepower.

Despite the Agenbroad simplified stove flow model, and other previous investigations, there is still a limited understanding of variations in the stove flow loss coefficient. Published contributions explicitly addressing access to the stove loss coefficient (also named the discharge coefficient) are very scarce. Table 1 summarizes some of the few papers dealing with the loss coefficient issue in stove modelling.

Following a common fluid mechanics approach, this loss coefficient is associated with an overall pressure drop through stove geometry. Thus, as a conclusion of the literature review in Table 1, losses in the flow field are supposed to be systematically characterized by empirical friction factors and single-valued head loss coefficients of different conduit components such as sudden contraction at inlet, friction loss in elbow length, loss due to friction in the pot gap zone, etc. This way of proceeding continues to appear in the eyes of many as the single rule to predict stove flow behaviour [11,26].

However, from a thermodynamics point of view, in addition to fluid friction, a real process can present other kinds of irreversibilities associated with heat-transfer mechanisms, mixing, chemical reactions, etc., and all resulting in the loss of process efficiency [27,28,29] related to the entropy-generation rate. Therefore, an energy devaluation (energy quality loss) manifests in a destruction of available work commonly known as exergy. Losses in the internal flow field of a technical application like energy conversion in a woodburning stove can from now be assumed to be losses of flow exergy. The notion on the quality of energy and its change during thermodynamic processes is today well addressed in many contributions, to name just a few, [30,31,32,33,34,35,36].

In recent years, the second law of thermodynamics in analyzing energy conversion in power-generating units permits a fresh look to evaluate some key features of the flow dynamics, heat transfer and chemical reactions through various systems. Many works dealing with entropy-generation analysis in flows involving heat transfer in natural convection processes can be found in the literature, e.g., [27,37]. These works concluded that the second law analysis plays a vital role in determining the frictional and heat-transfer losses [38]. Recently, refs. [39,40,41,42] and Schmandt [43,44] also analyzed the basic principles of entropy and its role in the momentum and heat transfer. However, these authors made an attempt to understand the physics beyond convective heat-transfer processes in an original way by introducing some alternative non-dimensional parameters that allow to also assess qualitative aspects during the energy-transfer processes.

To the best of our knowledge, no scientific paper has addressed woodburning stove engineering from the angle of the second law analysis. In researching this, not a single paper contains reference to words like entropy or exergy. Even when Agenbroad et al. [21,22] mentioned reduction of chimney effect due to a non-ideal heat addition profile assumption, the entropy or exergy concept was not dealt with in their model (see Table 1). Thus, it is not surprising to see that researchers in the cooking stove community focused exclusively on viscous and frictional losses when addressing the issue of stove flow resistance. The evaluation is being performed as if losses were occurring in an “isothermal cold flow field”, whereas heat-transfer processes are identified to be in turn important sources of irreversibilities [45].

The present contribution presents a new outlook on the stove flow loss coefficient assessment based on an exergetic analysis of the flowing fluid and making particular use of qualitative assessment numbers in energy-transfer processes. The study derives a simplified analytical model that permits to evaluate the entropy-generation rate due to steady-flow combustion and viscous dissipation in a natural draft shielded fire stove burning wood fuel. The effect of varying woodburning rate (or firepower) on the entropy-generation rate is assessed in a steady-state assumption. To validate this model, experiments have been conducted first using a G3300 cooking stove model without a cooking pot in place to better isolate the physical processes governing the intrinsic behaviour of the stove. Then, we referred to published literature [15,23,46] to confirm the practical case of a stove operating with a cooking piece in place.

The idea in carrying out this study is motivated by the observation, in the literature, of often erroneous results or simply the lack of information concerning the loss coefficient of cooking stoves. The loss coefficient is likely a determinant parameter in stove engineering. Without a comprehensive assessment method of this parameter, it will remain challenging to evaluate proper stove-operating behaviour. This work attempts to reconsider flow and heat transfer issues through a holistic approach.

This paper is organised as follows. The theoretical background along with the derivation of the loss coefficient is provided in Section 2. Since validation data shall be generated, the experimental setup together with the materials and methods utilized is introduced in Section 3. The obtained results and related discussions are presented in Section 4. The last Section 5 is devoted to conclusions.

## 2. Theoretical Formulation

This section provides the theoretical background in terms of thermodynamics and entropy-generation analysis. Thereby, the efficiency of the conversion process between thermal and mechanical energy is derived, and the loss coefficient is consistently provided.

### 2.1. Thermodynamics of Steady-Flow Combustion

#### 2.1.1. Conservation of Mass and Energy

Air flow along the chimney is due to buoyancy forces that drive hot gases upward. The mass balance for the combustion chamber in Figure 1 yields:

(1) $${\dot{m}}_{\mathrm{in}}+{\dot{m}}_{F}={\dot{m}}_{\mathrm{out}}$$

where ${\dot{m}}_{\mathrm{in}}$ is the cold air mass-flow rate entering the combustion chamber, ${\dot{m}}_{F}$ is the mass-burning rate of the fuel and ${\dot{m}}_{\mathrm{out}}$ is the exiting flue gas mass-flow rate.

Complete combustion of wood on a per-mole-of-fuel basis can be described by a generalized one-step overall reaction:

(2) $$C_{x}H_{y}O_{z}+\left(x+\frac{y}{4}-\frac{z}{2}\right)\left(O_{2}+3.76\;N_{2}\right)\to x\;CO_{2}+\frac{y}{2}\;H_{2}O+3.76\left(x+\frac{y}{4}-\frac{z}{2}\right)N_{2}$$

The first law of thermodynamics for a steady-flow combustion requires that the rate of heat transfer per mole of wood burnt balances the difference between the enthalpies of the reactant and product streams.

The expression for the total molar enthalpy is given as:

(3) $$Q_{m}=x\;{\bar{h}}_{f}^{{}^{\circ}}{,}_{CO_{2}}+\;\frac{y}{2}\;{\bar{h}}_{f}^{{}^{\circ}}{,}_{H_{2}O}-\;{\bar{h}}_{f}^{{}^{\circ}}{,}_{C_{x}H_{y}O_{z}}$$

The quantities ${\bar{h}}_{f}^{{}^{\circ}}{,}_{i}$ in Equation (3) individually represent the enthalpy of formation of the compound *i*. Note that the ${\bar{h}}_{f}^{{}^{\circ}}{,}_{i}$ for $O_{2}$ and $N_{2}$ are zero as they are all elementary substances.

The rate of total heat of combustion is linked to the low heating value (LHV) of the wood species as:

(4) $$\dot{Q}=a\cdot Q_{m}=a\cdot M\cdot LHV$$

where *a* represents the molar rate of wood consumption and *M* its molecular weight.

The total enthalpy in a compact form reduces to:

(5) $$\dot{Q}={\dot{m}}_{F}\cdot LHV$$

Part of this thermal energy takes on the form of flow energy in the flue gas (${\dot{Q}}_{\mathrm{flue}}$), which is responsible for the buoyant flow through the stove and past the surface of the pot (Figure 1). An in-depth analysis in references such as [11,15] shows that the rest of the energy identifies principally components of radiative heat transfer from char bed to the pot bottom and/or to the surrounding environment (${\dot{Q}}_{\mathrm{char}\;\mathrm{radiation}}$), heat loss from the stove through insulation (${\dot{Q}}_{\mathrm{heat}\;\mathrm{loss}}$), radiative heat loss through the feed opening (${\dot{Q}}_{\mathrm{door}\;\mathrm{loss}}$) and the heat loss due to evaporation and sensible heat of fuel moisture (${\dot{Q}}_{\mathrm{fuel}\;\mathrm{moisture}}$).

Applying the energy balance to the entire cooking stove:

(6) $$\dot{Q}={\dot{Q}}_{\mathrm{flue}}+{\dot{Q}}_{\mathrm{char}\;\mathrm{radiation}}+\;{\dot{Q}}_{\mathrm{heat}\;\mathrm{loss}}+{\dot{Q}}_{\mathrm{door}\;\mathrm{loss}}+{\dot{Q}}_{\mathrm{fuel}\;\mathrm{moisture}}$$

For simplicity, in the flame zone, air, wood volatiles and combustion gases can all be modeled as a single ideal gas. So, the enthalpy of reaction distributed over the air mass-flow rate ${\dot{m}}_{A}$ crossing the stove can be written:

(7) $${\dot{Q}}_{\mathrm{flue}}={\dot{m}}_{A}\cdot {\bar{c}}_{p}\cdot \left(T_{H}-T_{0}\right)$$

where ${\bar{c}}_{p}$ is a mean value of specific heat capacity at constant pressure, $T_{0}$ is the temperature of the ambient and $T_{H}$ is the exit flue gas temperature. This means that ${\bar{c}}_{p}$ is determined:

(8) $${\bar{c}}_{p}=\sum_{k=1}^{N}c_{pk}Y_{k}$$

where $c_{pk}$ are the specific heat capacities at constant pressure of species *k* and $Y_{k}$ is their respective mass fractions, for k=1 to *N*, and *N* is the number of species in the reacting mixture. As combustion of wood proceeds in excess air conditions, the thermophysical properties of air dominate and the mass heat capacity of the mixture is very close to that of air. The value of ${\bar{c}}_{p}$ for air changes only from 1000 to 1200 J·kg${}^{-1}$·K${}^{-1}$ when temperature varies from 300 to 1500 K, so that ${\bar{c}}_{p}$ can be considered with a good approximation to be constant [47]. As discussed in [21], comparing model-predicted mass-flow rate dependence with firepower using a polynomial in place of a constant specific heat capacity, the resulting error is relatively small and justifiable to neglect its variation. The next section derives the temperature equation in the combustion chamber.

#### 2.1.2. Temperature Equation

One needs a consistent temperature profile within the stove prior to addressing the entropy-generation rate equation in Section 2.2. To achieve this, some additional simplifying assumptions are needed:

- Geometry of the domain is assimilated to a vertical cylindrical chimney.
- Flow is considered to be laminar, uniform (steady) and one dimensional axisymmetric.
- Heat addition proceeds gradually along the height of the chimney, see [21,22].
- Thermodynamic properties of the flue gases are the same as those of air.
- Radiative heat transfer of the flue gases is negligible, less than 1% of the flame radiation heat balance on the energy balance of the entire stove, as reported by [11,15].

The temperature equation for the incompressible 1-D reacting flow in steady state can be found in e.g., [47] as:

(9) $$\rho V\frac{dT}{dx}=-\frac{k}{{\bar{c}}_{p}}\frac{d^{2}T}{dx^{2}}+\frac{{\dot{Q}}_{\mathrm{flue}}}{{\bar{c}}_{p}h_{c}}$$

In this transport equation, ρ is the air density, *V* the velocity, *k* the air thermal conductivity, $h_{c}$ the height of the combustion chamber and $\frac{{\dot{Q}}_{\mathrm{flue}}}{h_{c}}$ the volume unit enthalpy of reaction added to the flue gas.

The general form of the solution to the differential equation is given as:

(10) $$T\left(x\right)=C_{1}e^{-\frac{\rho {\bar{c}}_{p}V}{k}x}+\frac{{\dot{Q}}_{\mathrm{flue}}}{\rho {\bar{c}}_{p}Vh_{c}}x+C_{2}$$

The quantity $\frac{\rho {\bar{c}}_{p}Vx}{k}$ (or $\frac{Vx}{\alpha }$ with α the thermal diffusivity) in Equation (9) can be associated with the Peclet number Pe along the flow, which is defined as the ratio of the bulk flow heat transfer by convection to the heat transfer by conduction. Scaling analysis can show that for all the parameters under study Pe⩾400. Therefore, heat conduction as well as the first term in Equation (9) can be safely neglected.

Inserting the boundary condition $T\left(0\right)=T_{0}$ at the entrance, the temperature profile results in the same form as in [21] when assuming a uniform heat addition with rising flame:

(11) $$T\left(x\right)=T_{0}+\frac{{\dot{Q}}_{\mathrm{flue}}}{{\dot{m}}_{A}{\bar{c}}_{p}h_{c}}x$$

and its gradient:

(12) $$\frac{dT(x)}{dx}=\frac{{\dot{Q}}_{\mathrm{flue}}}{{\dot{m}}_{A}{\bar{c}}_{p}h_{c}}$$

The heat released from combustion entrains air circulation due to the density difference between cold air and hot flue gases. The basics of fluid dynamics applied to a cooking stove is introduced next.

#### 2.1.3. Energy of The Flowing Fluid

The rate of the total energy θ contained in the flowing fluid takes the form:

(13) $$\theta ={\dot{m}}_{A}\left[Pv+\left(u+e_{k}+e_{p}\right)\right]$$

where *P* is the fluid pressure, *v* the specific volume and *u* the specific internal energy, while $e_{k}$ and $e_{p}$ express the mass kinetic and the mass potential energy of the flow, respectively. The additional form of energy, the flow energy Pv, represents the energy needed to admit and evacuate the flow in the control volume [30].

Equation (13) can be rewritten taking into account the energy of the flow via the specific enthalpy, h=u+Pv, as:

(14) $$\theta ={\dot{m}}_{A}\left(h+\frac{V^{2}}{2}+gx\right)$$

From the integral form of Bernoulli’s equation, the fluid flow through the combustion chamber is determined by a momentum balance of airflow due to buoyancy and pressure losses through friction, bends, expansions and contractions in the flow path [9] as:

(15) $$\frac{\rho_{H}V_{H}^{2}}{2}=gh_{c}\left(\rho_{0}-\rho_{H}\right)-\sum_{l=1}^{N}\rho_{H}\frac{V_{H}^{2}}{2}\left(f_{l}\frac{x_{l}}{D_{h,l}}+K_{l}\right)$$

where *g* is the gravity constant, $V_{H}$ the hot gas velocity, $h_{c}$ the chimney height and $\rho_{0}$ and $\rho_{H}$, respectively, the ambient and the hot-gas density.

In subsonic combustion, as the flame speeds are small compared to the sound speed, pressure can be considered constant [47]. Thus, considering the ideal gas equation, the density change through the flame front can be directly related to the temperature change as:

(16) $$\frac{\rho_{0}}{\rho_{H}}=\frac{T_{H}}{T_{0}}$$

Using the continuity equation, the mass-flow rate provided by the buoyantly driven flow in the common form of the chimney effect is then given by Equation (17):

(17) $${\dot{m}}_{A}=CA\;\left(\frac{P}{RT_{H}}\;\right)\sqrt{2gh_{c}\left(\frac{T_{H}-T_{0}}{T_{0}}\right)}$$

where *C* is the loss coefficient, $T_{0}$ the temperature of the ambient and $T_{H}$ the hot gas temperature and *A* the flow cross-section area. *P* stands for the ambient pressure and *R* the perfect gas constant.

The loss coefficient is introduced to account for all inefficiencies in the chimney effect (0≤C≤1). In a practical sense:

(18) $$C=\frac{{{\dot{m}}_{A}}_{\;\mathrm{actual}}}{{{\dot{m}}_{A}}_{\;\mathrm{theoretical}}}$$

It would theoretically be 1 for an ideal cookstove with neither loss during the heat addition to kinetic energy conversion nor viscous dissipations. Indeed, it is debatable whether this could be possible; otherwise, what could be this limit value? The loss coefficient issue will be discussed in very wide and detailed terms in Section 4.

#### 2.1.4. Second Law and Exergy Balance of the Flowing Fluid

The entropy-generation rate arising along the flow stream crossing the boundaries of a control volume can be determined on a mass-flow rate basis as a net entropy balance taking into account the in- and the outflowing entropies as well as that transferred by energy flows in the form of heat into and out of the system.

For a steady flow, single stream, the entropy-generation rate is given by:

(19) $${\dot{S}}_{\mathrm{gen}}={\dot{m}}_{A}\left(s_{\mathrm{out}}-s_{\mathrm{in}}\right)-\sum_{k=1}^{N}\frac{{\dot{Q}}_{k}}{T_{k}}⩾0$$

where $s_{\mathrm{in}}$ and $s_{\mathrm{out}}$ are, respectively, the entropy per unit mass of flow entering the system and that of the flow exiting the system, and ${\dot{Q}}_{k}$ is the heat transferred through the boundary at temperature $T_{k}$ at location *k*.

The cooking stove combustion chamber acts like a producer of entropy as the heat transferred ${\dot{Q}}_{\mathrm{flue}}$ responsible for the buoyant flow discharges in it at a given temperature. Exergy is the precious part of this thermal energy which can be used by work until it is part of the internal energy of the ambient.

**Figure 2:**
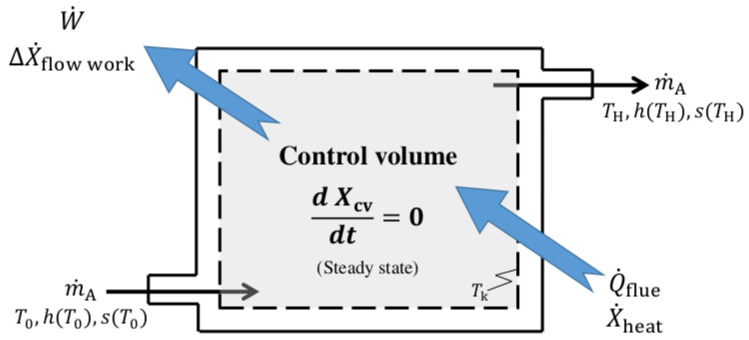
The rate of exergy change within the control volume ${\dot{X}}_{\mathrm{cv}}$ is equal to the rate of net exergy transfer through the control volume boundary by heat ${\dot{X}}_{\mathrm{heat}}$, work $\dot{W}$ and mass flow minus the rate of exergy destruction within the boundaries of the control volume. Note that in a steady state ${\dot{X}}_{\mathrm{cv}}$ is zero.

The exergy balance during the steady-flow combustion as sketched in Figure 2 takes into account:

1. The rate of exergy flow by heat transfer to the flue gases ${\dot{X}}_{\mathrm{heat}}$ that can be determined defining a Carnot factor $\eta_{c_{1}}$ which determines the quality of the heat depending on its temperature:
  (20) $${\dot{X}}_{\mathrm{heat}}=\eta_{c_{1}}\cdot {\dot{Q}}_{\mathrm{flue}}$$
  According to the linear temperature profile adopted in Section 2.1.2, flame can be modeled as a heat reservoir along stove chimney height that supplies heat indefinitely at temperatures gradually raising from $T_{0}$ to $T_{H}$. So care is taken to determine a mean Carnot efficiency by integration:
  (21) $$\eta_{c_{1}}=\frac{1}{T_{H}-T_{0}}\int_{T=T_{0}}^{T_{H}}\left(1-\frac{T_{0}}{T}\right)dT$$
  and
  (22) $$\eta_{c_{1}}=1-\frac{T_{0}}{T_{H}-T_{0}}\cdot \ln\frac{T_{H}}{T_{0}}$$
  The Carnot efficiency represents the fraction of the energy transferred from the heat source that can be converted to work, see Cengel [30].
  The rate of exergy flow by heat transfer becomes:
  (23) $${\dot{X}}_{\mathrm{heat}}=\left(1-\frac{T_{0}}{T_{H}-T_{0}}\cdot \ln\frac{T_{H}}{T_{0}}\right)\cdot {\dot{Q}}_{\mathrm{flue}}$$
  Otherwise, considering Equations (7) and (23), the difference $\left[\left(1-\eta_{c_{1}}\right)\cdot {\dot{Q}}_{\mathrm{flue}}\right]$ determines the rate at which exergy destruction due to heat transfer takes place:
  (24) $${{\dot{X}}_{\mathrm{destroyed}}}_{\left(\mathrm{heat}\right)}=\left(\frac{T_{0}}{T_{H}-T_{0}}\ln\frac{T_{H}}{T0}\right)\cdot {\dot{m}}_{A}{\bar{c}}_{p}\left(T_{H}-T_{0}\right)$$
  (25) $${{\dot{X}}_{\mathrm{destroyed}}}_{\left(\mathrm{heat}\right)}={\dot{m}}_{A}{\bar{c}}_{p}\cdot T_{0}\ln\frac{T_{H}}{T_{0}}$$
2. The rate of exergy change of the flow stream (exergy of the flowing fluid) across the combustion chamber that is written as:
  (26) $${\dot{X}}_{\mathrm{mass},\mathrm{in}}-{\dot{X}}_{\mathrm{mass},\mathrm{out}}={\dot{m}}_{A}\cdot \;\left[\left(h\left(T_{0}\right)-h\left(T_{H}\right)\right)-T_{0}\underset{N\mathrm{et}\;\mathrm{entropy}\;\mathrm{transfer}\;\mathrm{by}\;\mathrm{heat}\;\mathrm{and}\;\mathrm{mass}}{\underset{︸}{\cdot \left(s\left(T_{0}\right)-s\left(T_{H}\right)\right)}}+\frac{V_{H}^{2}}{2}+gh_{c}\right]$$
  where ${\dot{X}}_{\mathrm{mass},\mathrm{in}}$ is the rate of exergy transferred by mass when the mass in the amount of ${\dot{m}}_{A}$ enters the control volume and ${\dot{X}}_{\mathrm{mass},\mathrm{out}}$ is the rate of exergy evacuated by mass when the mass in the same amount leaves the control volume [30]. Mass flow into the system is accompanied by enthalpy $h(T_{0})$ and entropy $s(T_{0})$, and out of the system by $h(T_{H})$ and $s(T_{H})$, respectively. In Equation (26), $\frac{V_{H}^{2}}{2}$ and $gh_{c}$ are, respectively, exergy change associated with the mass kinetic and the mass potential energy of the flow.
3. The rate of exergy loss (or exergy destruction) of useful work by any other mechanisms at a location *k* (i.e., interaction of hot flue gases with the surface of the pot or with inner stove chimney surface) that is given:
  (27) $${\dot{X}}_{\mathrm{destroyed},\;k}=T_{0}\cdot {\dot{S}}_{\mathrm{gen},\;k}$$
  directly proportional to the rate of entropy generation ${\dot{S}}_{\mathrm{gen},k}$ in this form of relation known as the Gouy–Stodola theorem [31].

Finally, neglecting exergy of kinetic energy and exergy of potential energy (compared to other terms in Equation (26)), the exergy balance of the flowing fluid is summarized as:

(28) $$\left(1-\frac{T_{0}}{T_{H}-T_{0}}\cdot \ln\frac{T_{H}}{T_{0}}\right)\cdot {\dot{Q}}_{\mathrm{flue}}+{\dot{X}}_{\mathrm{mass},\mathrm{in}}-{\dot{X}}_{\mathrm{mass},\mathrm{out}}-{\dot{X}}_{\mathrm{destroyed},\;k}=0$$

Otherwise, the energy flux applied as flow work $\Delta {\dot{X}}_{\mathrm{flow}}$ can be written:

(29) $$\Delta {\dot{X}}_{\mathrm{flow}}={\dot{X}}_{\mathrm{mass},\mathrm{out}}-{\dot{X}}_{\mathrm{mass},\mathrm{in}}=\;\eta_{c_{1}}\cdot {\dot{Q}}_{\mathrm{flue}}-{\dot{X}}_{\mathrm{destroyed},\;k}$$

Equation (29) is an important result in the analysis of convective heat transfer. This relation states that the energy flux applied as flow work is pure exergy which is lost in consecutive dissipation processes.

**Figure 3:**
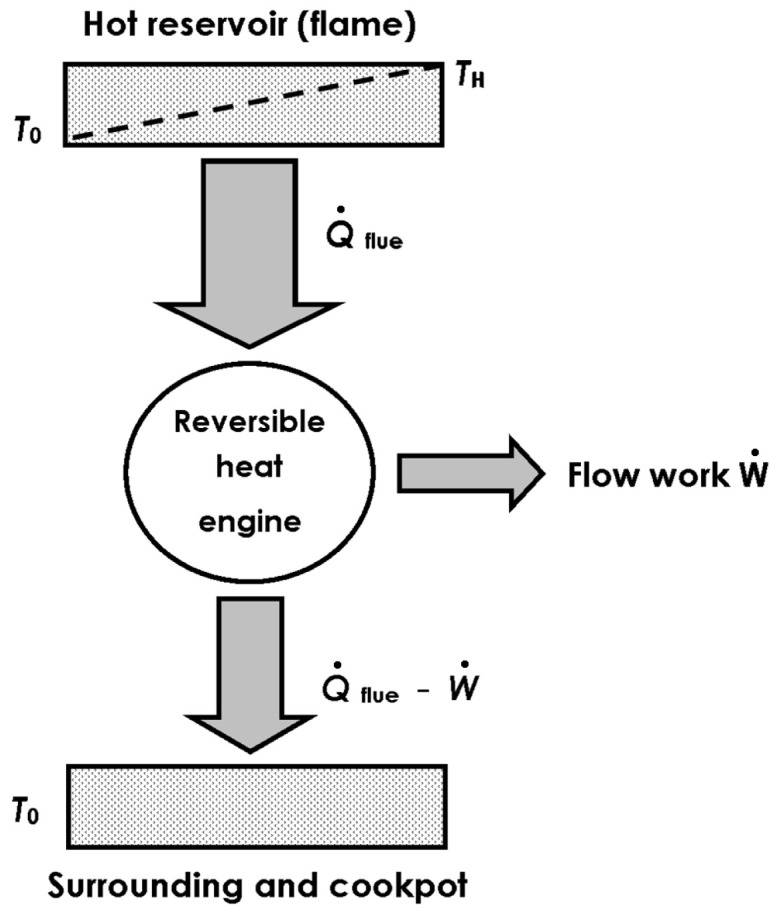
Open thermodynamic system in steady state equivalent to a reversible heat engine that operates between hot reservoir (flame) and atmospheric temperatures. The engine releases its work output into flow work, and rejects heat to the pot and to the surrounding environment.

Figure 3 shows the thermodynamic system equivalent to the simplified cooking stove model.

#### 2.1.5. Alternative Assessment Numbers in Energy Transfer Processes

As mentioned before, the quantity of energy is conserved, but its quality deteriorates during energy-transfer processes according to Equation (29). When thermodynamic considerations are added to the analysis and interpretation of convective heat-transfer situations, it turns out that the heat-transfer coefficient *h* or the Nusselt number Nu are no more precise parameters to access qualitative aspects of the energy-transfer processes, see [42]. In [39,48] are introduced some alternative non-dimensional parameters for a comprehensive characterization of the energy devaluation chain consecutive to the unit-transfer operations.

##### Energy Devaluation in Heat-Transfer Processes

The *energy-devaluation number $N_{i}$* for an energy-transfer operation *i* indicates how much of the *entropic potential* is used:

(30) $$N_{i}=\frac{T_{0}\cdot {\dot{S}}_{\mathrm{gen},i}}{\dot{E}}$$

where ${\dot{S}}_{\mathrm{gen},i}$ the entropy-generation rate reported to the unit-transfer operation *i*. This entropy generation is seen in the context of the devaluations of the energy-transfer rate $\dot{E}$ that happened prior to the-transfer operation *i* and that will happen afterwards.

##### Losses Due to Dissipation of Mechanical Work

Specifically, in a convective heat-transfer process, flow work rate is needed to maintain the flow into which heat transfer occurs. So a second coefficient is needed which is defined as the *exergy destruction number* $N^{E}$, indicating the loss of exergy in the flow field:

(31) $$N^{E}=\frac{T_{0}\cdot {\dot{S}}_{\mathrm{gen},D}}{\dot{E}}$$

where ${\dot{S}}_{\mathrm{gen},D}$ is the entropy dissipation rate due to dissipation of mechanical energy and $\dot{E}$ the kinetic energy.

Take care that in $N^{E}$ it is the kinetic energy of the fluid flow which is used as a reference quantity, whereas $N_{i}$ refers to the quantity of energy or heat transferred. However, it is not the kinetic energy that is devaluated but the energy that enters the system as flow work [39].

##### Overall Exergy Devaluation in Heat-Transfer Processes

For an overall assessment of a convective heat-transfer process, Herwig [39] refers subtly to the sum of exergy losses (in the temperature and in the flow field) to the exergy transferred in the process, which is the product $\eta_{c_{2}}\cdot {\dot{Q}}_{c-p}$. The *overall exergy loss number* reads:

(32) $${\hat{N}}^{E}=\frac{T_{0}\cdot \left({{\dot{S}}_{\mathrm{gen}}}_{\left(\mathrm{heat}\right)}\;+{{\dot{S}}_{\mathrm{gen}}}_{\left(\mathrm{viscous}\right)}\right)}{\eta_{c_{2}}\cdot {\dot{Q}}_{c-p}}$$

In the equation above, the term $\eta_{c_{2}}$ is the Carnot factor for the consecutive convective exergy-transfer process different from $\eta_{c_{1}}$ which previously defined the exergy part of the energy transferred after combustion to the flue gases. The physical meaning of the Carnot factor $\eta_{c_{2}}$ will be resumed in Section 2.2.2.

The measure for the quality of energy and its potential degradation in energy-transfer processes is entropy. In the next section we developed a simplified analytical expression of the total entropy-generation rate in the flowing fluid due to heat-transfer processes and viscous dissipations.

### 2.2. Entropy Generation Rate: Analytical Solution

#### 2.2.1. Stove Operating without Cooking Pot

The infinitesimal change of the rate of entropy generation is:

(33) $$d{\dot{S}}_{\mathrm{gen}}={\dot{m}}_{A}ds-\frac{\delta {\dot{Q}}_{c-w}}{T_{g}}$$

where ${\dot{Q}}_{c-w}$ is the rate of convective heat transferred from gases to the inner combustion chamber walls at temperature $T_{g}$.

In a steady-state regime for an insulated stove and considering the radiative heat transfer of the flame to be negligible [11,15], Equation (33) can be simplified to:

(34) $$d{\dot{S}}_{\mathrm{gen}}\approx {\dot{m}}_{A}ds$$

By virtue of the principle of conservation of energy, the infinitesimal mass sensible enthalpy increase dh in the flue gases and according to Equation (7) denotes:

(35) $$dh={\bar{c}}_{p}dT$$

The Gibbs–Duhem relation corresponding to the energetic fundamental relation is given:

(36) Tds=dh−vdp

where *v* stands for the specific volume and *p* the pressure.

Rearranging Equations (34) and (35) in (33) gives:

(37) $$d{\dot{S}}_{\mathrm{gen}}={\dot{m}}_{A}\cdot \left(\frac{{\bar{c}}_{p}dT}{T}-\frac{vdp}{T}\right)$$

and expressing the specific volume $v=\rho^{-1}$

(38) $$d{\dot{S}}_{\mathrm{gen}}={\dot{m}}_{A}\cdot \left(\frac{{\bar{c}}_{p}dT}{T}-\frac{1}{\rho }\frac{dp}{T}\right)$$

Let us now introduce derivations with respect to *x* (the spatial coordinate):

(39) $$\frac{d{\dot{S}}_{\mathrm{gen}}}{dx}={\dot{m}}_{A}\cdot \left(\frac{{\bar{c}}_{p}}{T(x)}\frac{dT(x)}{dx}-\frac{1}{\rho T(x)}\frac{dp}{dx}\right)$$

In a rearranged form, integration along the height of the combustion chamber gives:

(40) $${\dot{S}}_{\mathrm{gen}}={\dot{m}}_{A}{\bar{c}}_{p}\cdot \int_{x=0}^{h_{c}}\frac{1}{T(x)}\frac{dT(x)}{dx}dx-{\dot{m}}_{A}\cdot \int_{x=0}^{h_{c}}\frac{1}{\rho T(x)}\frac{dp}{dx}dx$$

In the second term of the right-hand side of the expression above, the pressure drop $-\frac{dp}{dx}$ evaluated on a finite distance can be related to the dynamic pressure as:

(41) $$-\frac{\Delta p}{\Delta x}=K\rho \frac{V^{2}}{2x}$$

taking into account the definition of the chimney effect, see Equation (16):

(42) $$-\frac{\Delta p}{\Delta x}=gK\rho \frac{T\left(x\right)-T_{0}}{T_{0}}$$

Note that the single *K*-value represents a total heat loss through the stove related to viscous dissipations in the fluid flow, see Equation (15).

Therefore, entropy-generation rate becomes explicitly related to mass-flow rate and temperature:

(43) $${\dot{S}}_{\mathrm{gen}}={\dot{m}}_{A}{\bar{c}}_{p}\cdot \int_{x=0}^{h_{c}}\frac{1}{T(x)}\frac{dT(x)}{dx}dx+{\dot{m}}_{A}\cdot \int_{x=0}^{h_{c}}\frac{gK\left(T\left(x\right)-T_{0}\right)}{T\left(x\right)T_{0}}dx$$

(44) $${\dot{S}}_{\mathrm{gen}}={\dot{m}}_{A}{\bar{c}}_{p}\cdot \int_{x=0}^{h_{c}}\frac{1}{T(x)}\frac{dT(x)}{dx}dx+{\dot{m}}_{A}\cdot \int_{x=0}^{h_{c}}gK\cdot \left(\frac{1}{T_{0}}-\frac{1}{T(x)}\right)\cdot dx$$

Substituting in Equation (44) the temperature profile T(x) from Equation (11) and its derivative with respect to *x* from Equation (12):

(45) $${\dot{S}}_{\mathrm{gen}}={\dot{m}}_{A}{\bar{c}}_{p}\cdot \int_{x=0}^{h_{c}}\frac{{\dot{Q}}_{\mathrm{flue}}}{{\dot{m}}_{A}h_{c}\left(T_{0}+\frac{{\dot{Q}}_{\mathrm{flue}}}{{\dot{m}}_{A}{\bar{c}}_{p}h_{c}}x\right)}dx+{\dot{m}}_{A}\cdot \int_{x=0}^{h_{c}}\frac{gK}{T_{0}}dx-{\dot{m}}_{A}\cdot \int_{x=0}^{h_{c}}gK\cdot \frac{1}{T_{0}+\frac{\left(T_{H}-T_{0}\right)}{h_{c}}x}dx$$

Likewise, considering the expression of the heat addition ${\dot{Q}}_{\mathrm{flue}}$ in Equation (7):

(46) $${\dot{S}}_{\mathrm{gen}}={\dot{m}}_{A}{\bar{c}}_{p}\cdot \int_{x=0}^{h_{c}}\frac{T_{H}-T_{0}}{T_{0}h_{c}+\left(T_{H}-T_{0}\right)x}dx+{\dot{m}}_{A}\cdot \int_{x=0}^{h_{c}}\frac{gK}{T_{0}}dx-{\dot{m}}_{A}\cdot \int_{x=0}^{h_{c}}gK\cdot \frac{1}{T_{0}+\frac{\left(T_{H}-T_{0}\right)}{h_{c}}x}dx$$

Finally, the entropy generation in the flow stream that results from the heat transfer and frictional pressure drop processes is:

(47) $${\dot{S}}_{\mathrm{gen}}=\underset{{{\dot{S}}_{\mathrm{gen}}}_{\left(\mathrm{heat}\right)}}{\underset{︸}{{\dot{m}}_{A}{\bar{c}}_{p}\cdot \ln\frac{T_{H}}{T_{0}}}}+\underset{{{\dot{S}}_{\mathrm{gen}}}_{\left(\mathrm{viscous}\;\mathrm{and}\;\mathrm{frictional}\;\mathrm{pressure}\;\mathrm{drop}\right)}}{\underset{︸}{K{\dot{m}}_{A}gh_{c}\cdot \left(\frac{1}{T_{0}}-\frac{1}{T_{H}-T_{0}}\cdot \ln\frac{T_{H}}{T_{0}}\right)}}$$

The first term on the right-hand side represents the entropy-generation rate due to heat transfer. Note that this term multiplied by $T_{0}$ matches the expression of the exergy-destruction rate due to heat transfer in Equation (25). The second term represents the contributions due to viscous processes.

(48) $${\dot{S}}_{\mathrm{gen}}={\dot{m}}_{A}{\bar{c}}_{p}\cdot \left[\ln\frac{T_{H}}{T_{0}}+K\frac{gh_{c}}{{\bar{c}}_{p}T_{0}}\cdot \left(1-\frac{T_{0}}{T_{H}-T_{0}}\cdot \ln\frac{T_{H}}{T_{0}}\right)\right]$$

An entropy-generation number $N_{s}$ introduced by Bejan [49] can be defined in a dimensionless form as:

(49) $$N_{s}=\frac{{\dot{S}}_{\mathrm{gen}}}{{\dot{m}}_{A}{\bar{c}}_{p}}=\ln\frac{T_{H}}{T_{0}}+K\frac{gh_{c}}{{\bar{c}}_{p}T_{0}}\cdot \left(1-\frac{T_{0}}{T_{H}-T_{0}}\cdot \ln\frac{T_{H}}{T_{0}}\right)$$

Considering the expression of Carnot efficiency in Equation (21):

(50) $$N_{s}=\ln\frac{T_{H}}{T_{0}}+\eta_{c_{1}}\cdot \frac{gh_{c}}{{\bar{c}}_{p}T_{0}}\cdot K$$

It appears that the entropy-generation rate due to viscous dissipations is directly related to the exergy Carnot factor $\eta_{c_{1}}$. The dimensionless quantity $\frac{gh_{c}}{{\bar{c}}_{p}T_{0}}$ is also known as the Gebhart number, accounting for the viscous dissipation of thermal energy in natural convection processes [37]. The single *K*-value represents a total heat-loss coefficient associated with the conduit components. Thus, the Carnot factor plays the role of a weighting parameter for the potential to generate entropy by frictional pressure-drop effects. The higher the flue gas temperature, the higher the exergy flow rate, and the more viscous dissipations are accounted for.

#### 2.2.2. Stove Operating with Cooking Pot

Let us consider now the practical case of the stove operating with a cooking pot containing, let us say, a given quantity of water. Hot flue gases interact with the outer surface of the pot. Therefore, this convective heat transfer contributes to the destruction of exergy flow.

The rate of thermal energy transfer to pot ${\dot{Q}}_{c-p}$ is affected by the convective coefficient *h* changing with the mass-flow rate, the pot exposed surface area $A_{p}$ and the difference between the gases temperature $T_{g}$ and the averaged pot surface temperature $T_{p}$:

(51) $${\dot{Q}}_{c-p}=h\cdot A_{p}\cdot \left(T_{g}-T_{p}\right)$$

The convective coefficient is related to the Nusselt number:

(52) $$h=\frac{Nu\cdot k}{D}$$

where *k* is the thermal conductivity of the flue gas and *D* is the value of the chimney diameter.

The thermal energy transfer to pot can be rewritten:

(53) $${\dot{Q}}_{c-p}=\frac{Nu\cdot k\cdot A_{p}\cdot \left(T_{g}-T_{p}\right)}{D}$$

Zube [15] proposed an average Nu for a fully developed free jet impinging on a flat plate:

(54) $$Nu=0.565\cdot Pr^{0.5}\cdot Re^{0.5}$$

A constant Prandtl number Pr of air is assumed to be around 0.7 at 1 atm in the range of temperature between 300 K and 1500 K. The Reynolds number Re is written:

(55) $$Re\equiv \frac{\rho VD}{\mu (T)}$$

where ρ is the air density, μ(T) the air dynamic viscosity function of temperature, *V* the velocity of the fluid and *D* the diameter of the cylindrical stove chimney. The dependence of the stove flow Reynolds number on stove operation can be determined in the function of the mass-flow rate and the cross-section area *A* of the stove chimney as shown:

(56) $$Re\equiv \frac{\rho VDA}{\mu (T)A}=\frac{2\;{\dot{m}}_{A}}{\mu \left(T\right)\cdot \sqrt{A\pi }}$$

**Figure 4:**
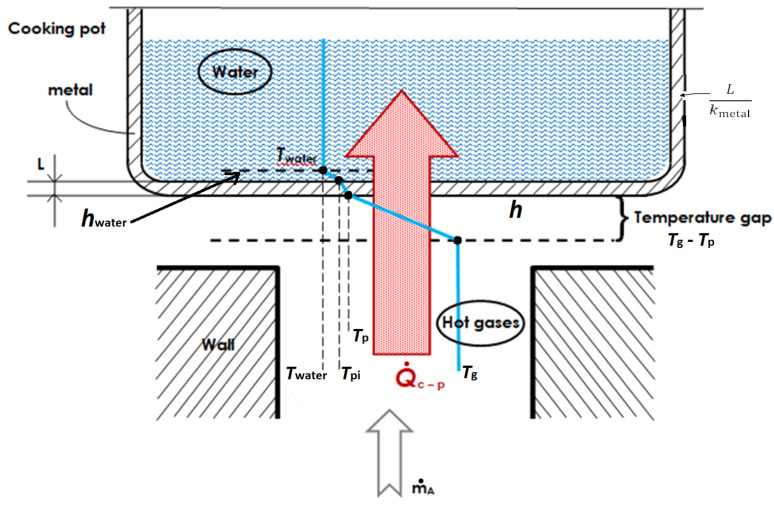
Hot gases at temperature $T_{g}$ convect a certain amount of energy ${\dot{Q}}_{c-p}$ to the external pot surface at temperature $T_{p}$. Then the heat is conducted through the metal (pot) of small thickness and finally convected from the internal surface of the pot at temperature $T_{\mathrm{pi}}$ into water at temperature $T_{\mathrm{water}}$.

The convective heat transfer sketched in Figure 4 reveals a “temperature gap” between the flame and the exposed surface of the pot. The heat-transfer interaction ${\dot{Q}}_{c-p}$ across this space remains undiminished [31].

The pot surface temperature $T_{p}$ can be determined by defining the overall heat-transfer coefficient *U* between the three media sketched in Figure 4:

(57) $$U=\frac{{\dot{Q}}_{c-p}}{A_{p}\cdot \left(T_{g}-T_{g}\right)}$$

The electrical analogy of resistance gives means to calculate *U*:

(58) $$U=\frac{1}{\frac{1}{h}+\frac{L}{k_{\mathrm{metal}}}+\frac{1}{h_{\mathrm{water}}}}$$

where *h* is the convective heat-transfer coefficient in the “temperature gap” between hot gases and external cooking pot surface, $k_{\mathrm{metal}}$ is the thermal conductivity of the metal (often aluminium) and *L* is its thickness and $h_{\mathrm{water}}$ is the free convection heat-transfer coefficient between internal pot surface and water. The order of magnitude of the heat-transfer coefficient is around 200 W·m${}^{-2}$·K${}^{-1}$ for *h*, $\frac{1}{160,000}$ W·m${}^{-2}$·K${}^{-1}$ for $\frac{L}{k_{\mathrm{metal}}}$ and around 5000 W·m${}^{-2}$·K${}^{-1}$ for $h_{\mathrm{water}}$ [50]. It can be seen that conduction resistance through the metal pot and internal surface convective resistance to water are negligible. The overall heat-transfer coefficient *U* becomes almost equal to the convective heat-transfer coefficient in the temperature gap *h*; therefore, $T_{p}\approx T_{\mathrm{water}}$.

Thus, the entropy-generation rate in this temperature gap can be written:

(59) $${{\dot{S}}_{\mathrm{gen}}}_{\left(c-p\right)}=\frac{{\dot{Q}}_{c-p}}{T_{p}}-\frac{{\dot{Q}}_{c-p}}{T_{g}}={\dot{Q}}_{c-p}\cdot \frac{\left(T_{g}-T_{p}\right)}{T_{g}T_{p}}$$

The loss of exergy as a result of this irreversibility is:

(60) $${{\dot{X}}_{\mathrm{destroyed}}}_{\left(c-p\right)}=T_{0}\cdot {{\dot{S}}_{\mathrm{gen}}}_{\left(c-p\right)}=\frac{T_{0}}{T_{p}}\left(1-\frac{T_{p}}{T_{g}}\right)\cdot {\dot{Q}}_{c-p}=\eta_{c_{2}}\cdot {\dot{Q}}_{c-p}$$

Caution must be taken when analyzing consecutive energy-transfer operations. The very question largely discussed in [48] is how to put energy-transfer assessment in the right perspective. Section 4 will address the way to deal with alternative non-dimensional parameters in order to assess adequately the overall energy devaluation in the present application.

## 3. Experimental Setup, Materials and Methods

In our experimental part, tests have been conducted solely for the basic case of stove without cooking pot. Given the complexity, validation for the practical case with pot in place refers to calculations and data in [15,22,46].

The two properties that most predominantly characterize the flow are mass-flow rate and temperature. The task is to assess the entropy-generation rate and associated quantities by means of the mass-flow rate and exhaust-gas temperature measured when the cooking stove is tested at different operating firepower levels.

In practice, differing firepower level is achieved by varying by hand the fuel-feed rate, the mass of fuel in the combustion chamber and fuel spacing [20]. Furthermore, though the actual fire is an intrinsically transient phenomenon, the stove will be considered to operate under steady state conditions by averaging its temporal behaviour. The next paragraphs present successively the stove, the fuel properties and the experimental protocol.

### 3.1. The Stove

**Figure 5:**
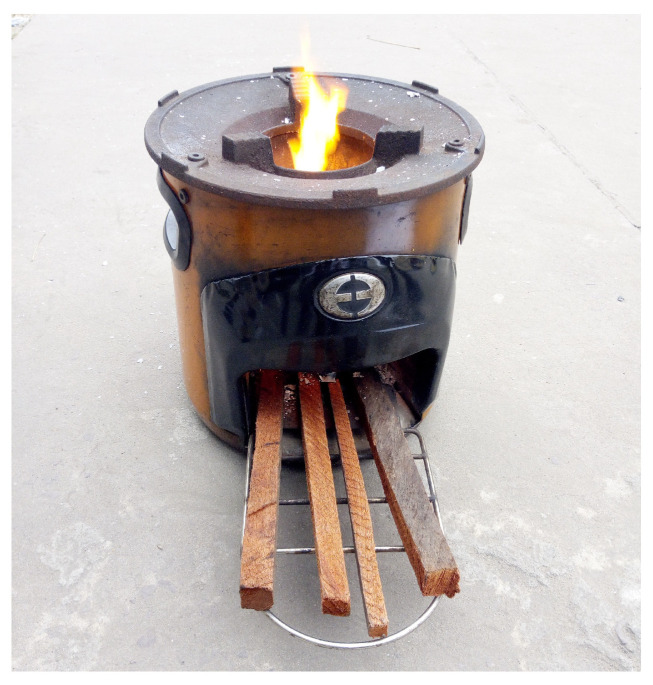
G3300 envirofit cookstove model.

Environfit G3300 in Figure 5 is a stick burning wood fuel cookstove, developed on the basis of the rocket elbow principle by Envirofit International, Inc. ( Fort Collins, CO, USA) (http://www.envirofit.org/ (accessed on 8 March 2020)). Many papers related to this model have been published by researchers at the Colorado State University [15,22,23,51]. Table 2 gives the G3300 geometrical parameters.

### 3.2. Wood Properties and Preparation

The wood used for this experiment is Entandrophragma Cylindricum (Sapele), a tropical species widely found in many African regions and commonly known as red wood because of its reddish tint. Table 3 gives the elemental analysis of Sapele from the literature report [52].

The wood is moderately heavy, with a density of 560–750 kg·m${}^{-3}$ at 12% moisture content. Chemical analysis from [53] shows that Sapele wood is slightly alkaline (pH = 8) and the net-heat of combustion of Sapele in the air-dry state (8% relative humidity) was measured to be 17.1 MJ·kg${}^{-1}$ and one can infer for an oven-dried sample a low heating value of 18.8 MJ·kg${}^{-1}$ [54]. To improve repeatability, Sapele wood in all our tests was used oven dried. Wood cribs of square and rectangular cross-sections were prepared: 2.5 × 2.5 × 33 cm (stick) and 1.3 × 2.5 × 33 cm ($\frac{1}{2}$ stick). Typical sample stick and $\frac{1}{2}$ stick (half) weighing, respectively, 96 and 48 g presented specific area ratios of 240 and 320 m${}^{-1}$, the calculation being limited to only the tip of long pieces of wood inserted a small distance (≈2.5 cm) into the fire.

### 3.3. Testing Protocol

Experiments have been performed using the Environfit G3300 wood cookstove under a hood. Each test consisted of 15 min data sampling periods over which firepower is held as constant as possible. Temporal recording of fuel mass reduction, bulk flow temperature and O${}_{2}$ concentration in the exhaust gas sample was also simultaneously measured. Temporal averaging was then applied to the 15 min data samples giving the values used for different firepower sample points. The sampling periods were counted from the time when the firepower reached approximately steady state behaviour excluding start-up and shut-down periods [20]. Data averaging was performed using GNUPLOT version 5.2 patchlevel 2.

#### 3.3.1. Measuring Fuel-Mass-Burning Rate and Firepower

The mass-burning rate of fuel was calculated as in [46] by keeping the stove on a sufficiently robust balance. Time intervals for every 0.01 kg fuel reduction were noted down for every batch of wood burned, while an experienced operator tries to maintain flame intensity as constant as possible. The average mass-burning rate of fuel was determined as the ratio of 0.01 kg to the average time for a set of readings $t_{\mathrm{avg}}$:

(61) $$\dot{m_{F}}=\frac{0.01}{t_{\mathrm{avg}}}$$

Operating firepower was calculated using expression Equation (5). Table 4 presents batch load characteristics, the stack giving wood cribs composition, the entrance area allowed by the stacking of wood cribs, the inlet area ratio (IAR) defined as the ratio of the cross-sectional area unoccupied by the wood at the feed door to the total entrance area [46] and the averaged fuel-mass-burning rate that resulted following pseudo-steady state firepower levels.

#### 3.3.2. Measuring %O${}_{2}$ and Calculating Mass-Flow Rate

The O${}_{2}$ concentration in the exhaust gas sample was determined via the syngas analyser GASBOARD-3100P of Cubic-Ruiyi Instrument based on ECD, where a fraction of the total flow is drawn by a suction pump through a sample line to the real-time (0.5 Hz) sensor.

The air mass-flow rate is calculated with the method in [21,22] using the stack exhaust volumetric %*O*${}_{2}$ instead of flow meters. This simplified exhaust %*O*${}_{2}$ approach is worth being exposed here again.

Combustion of wood in an excess of air can be described by the generalized form one-step reaction:

(62) $$aC_{x}H_{y}O_{z}+b\left(O_{2}+3.76N_{2}\right)\to dCO_{2}+eH_{2}O+fO_{2}+b\left(3.76N_{2}\right)$$

where *a*, *b*, *d*, *e* and *f* correspond to the stoichiometric coefficients expressed in molar rate units.

Stack exhaust %*O*${}_{2}$ is related to the overall one-step reaction summarized in Equation (61) as shown in Equation (62). The molar rate of oxygen (*b*) can be calculated as shown in Equation (63), (*b*) the function of the molar rate of fuel consumption (*a*), %*O*${}_{2}$ concentration and the elemental composition of the fuel (x=4.6, y=4.7, z=2.5).

(63) $$\%O_{2}=\frac{f\cdot 100}{d+e+f+3.76b}$$

(64) $$b=\frac{a(z-2x-\frac{y}{2})}{\%O_{2}\cdot 4.76-100}\times \left(50-\frac{\%O_{2}}{2}-\%O_{2}\cdot ax\right)$$

At the end, the mass-flow rate of air is obtained by multiplying (*b*) by the molecular weight, as shown in Equation (64).

(65) $${\dot{m}}_{A}=b\frac{\mathrm{kgmol}}{s}\cdot \left(32+3.76\cdot 28\right)\frac{\mathrm{kg}}{\mathrm{kgmol}}$$

#### 3.3.3. Measuring Temperature

Bulk flow temperature is measured using a K-type thermocouple placed at the approximate center of the chimney about 1 cm above the chimney exit.

## 4. Results: Validation and Discussion of the Second Law Approach

### 4.1. Stove without Pot

The thermal properties of air described by quantities including specific enthalpy and entropy at different temperatures are given in thermodynamic tables, for example, in [55]. Table 5 outlines the experimental values of time-averaged exiting gas temperature (column 4) and air mass-flow rate (column 6) at different fuel burning rates for the G3300 stove operating without cooking pot. The corresponding firepower levels were obtained according to Equation (5). Oxygen percentage values enter air mass-flow rate calculations following Equations (62)–(64). Experimental mass-flow rates and exit gas temperatures permit calculation of the stove flow Reynolds number using Equation (56). Values of Re vary in the interval between 438 and 1358. These low Reynolds numbers verify the initial hypothesis that the flow encountered is laminar.

Air mass-flow rates along with specific entropy changes (column 7) were used as inputs to calculate the total entropy-generation rate ${\dot{S}}_{\mathrm{gen}}$ appearing in the last column of Table 5. From this simplified model, it appears that for all the parameters under study, the contribution of the entropy-generation rate due to viscous dissipation in Equation (48) is less than 0.1% compared to the total entropy-generation rate. Following Gebhart [37] the dimensionless number $\frac{gh_{c}}{{\bar{c}}_{p}T_{0}}$ in Equation (48) shows that the effects of viscous dissipation in natural convection is appreciable indeed when the induced kinetic energy becomes appreciable compared to the amount of heat transferred. This occurs when either the equivalent body force is large (g) or when the convection region $\left(h_{c}\right)$ is extensive, but this is not the case here. This quantity remains of the order of $10^{-5}$ and therefore the effects of viscous dissipations can be safely ignored; this was the same as assumed in [38] citing earlier works devoted to the role of irreversibility distribution ratio on the total entropy generation. That said, it can be concluded that the major source of irreversibilities in fluid flow through a stove is in the heat-transfer process. In contrast, entropy generation due to viscous dissipation and fluid friction is negligible. Hence, for the rest of discussion, entropy due to viscous dissipation will not be shown as a separate component.

**Figure 6:**
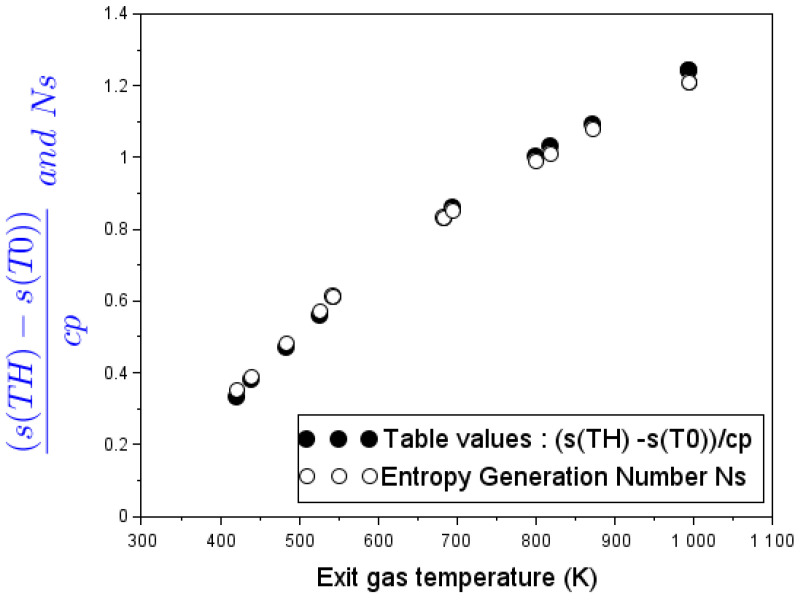
Specific-entropy-to-specific-heat ratio on the basis of measures on the G3300 stove operating without cooking pot and model-predicted dimensionless entropy number $N_{s}$.

Figure 6 depicts the evolution of the dimensionless entropy-generation number $N_{s}$ with respect to the exhaust-gas temperature. Considering Equation (49), the model-predicted $\left(\ln\frac{T_{H}}{T_{0}}\right)$ sample points obtained from experiments at different operating firepower levels agree well with thermodynamic table values of the specific entropy balance into and out of the combustion chamber reported to the specific heat as $\left(\frac{s\left(T_{H}\right)-s\left(T_{0}\right)}{{\bar{c}}_{p}}\right)$.

Table 6 gives the rate of the total energy variation of the flowing fluid Δθ in column 4, which is almost equivalent to the rate of sensible enthalpy increase obtained using Equations (7) and (13). Column 5 gives the rate of exergy destroyed ${{\dot{X}}_{\mathrm{destroyed}}}_{\left(\mathrm{heat}\right)}$ calculated by means of Equation (25). Note that the difference between the two corresponds approximately to the rate of the flow exergy balance $\Delta {\dot{X}}_{\mathrm{flow}}$ taking place according to Equation (29).

**Figure 7:**
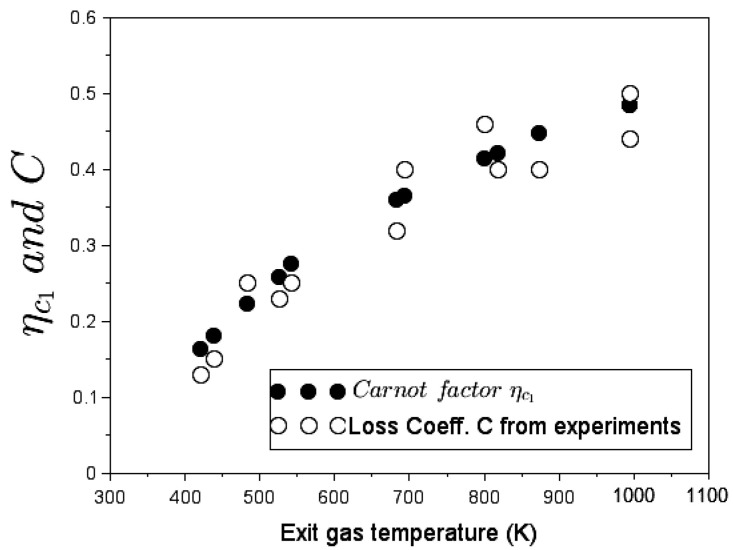
The Carnot factor $\eta_{c_{1}}$ and the loss coefficient *C* in function of the flue gas temperature for the G3300 stove without pot.

Now let us examine the link between the two energy conversion determinants, namely the loss coefficient *C* and the Carnot factor $\eta_{c_{1}}$. Equation (17) permits to equate experimental values of *C* for the G3300 woodburning stove operating without pot. On the other hand, Equation (22) completes Table 6 with values of the Carnot factor $\eta_{c_{1}}$ obtained by integration along the chimney height. It is important to note that the ratio of flow exergy rate $\Delta {\dot{X}}_{\mathrm{flow}}$ to total energy of the flowing fluid Δθ leads to the same Carnot factor results. Thus, Figure 7 plots the loss coefficient *C* and the Carnot factor $\eta_{c_{1}}$ sample points obtained at different exhaust-gas temperatures. This figure shows that $\eta_{c_{1}}$ and *C* trend overlay.

It appears in Figure 7 that the loss coefficient coincides remarkably with the Carnot factor that is identified as a measure of the quality of heat transfer. By definition, the Carnot factor $\eta_{c_{1}}$ defines the exergy part of the energy transferred. Thus, according to values in Table 6, when the cooking stove operates at low firepower levels (e.g., firepower = 0.5 kW and exit gas temperature = 421 K), a big amount of heat of combustion (≈85%) is not available in the form of useful (potential) work, in the sense that it does not participate in generating fluid motion, whereas at higher firepower levels (e.g., 5.9 kW and 994 K), almost half of the primary energy contributes to flow work and only half of the energy content is degraded.

In the basic case of the cookstove running without a cooking piece, the energy that enters the system as flow work is pure exergy that subsequently devaluates by dissipation processes according to Equation (29). The second case of the stove equipped with a cooking pot will show how flue gases and pot interaction degrade this flow work potential.

### 4.2. Stove with Cooking Pot

Contrary to the preceding case, in a stove operating with a pot in place, two energies are subjected to degradation in the convective heat-transfer process (the transferred thermal energy and the needed flow work). Table 7 illustrates the calculation results for mass-flow rate ${\dot{m}}_{A}$, hot gas temperature impinging the bottom surface of the pot $T_{g}$ (column 3) for the one-door rocket stove at different firepower levels in Zube [15].

The exit gas temperature $T_{\mathrm{exit}}$ (column 4) can be determined with the function of the rate of heat transfer to pot ${\dot{Q}}_{c-p}$ in a rearrangement of the first law of thermodynamics:

(66) $$T_{\mathrm{exit}}=T_{g}-\frac{{\dot{Q}}_{c-p}}{{\dot{m}}_{A}{\bar{c}}_{p}}$$

The hot gas temperature falls down from the exposed pot bottom surface to the exit port, as upward flows transport *exergy* to the pot. Then, the final water temperature inside the pot for each test can be also obtained in this way:

(67) $$T_{\mathrm{water}}=\frac{\dot{Q}\cdot t_{\mathrm{test}}\cdot \eta_{\mathrm{th}}}{m_{\mathrm{water}}\cdot {\bar{c}}_{p_{\mathrm{water}}}}+T_{0}$$

where $\dot{Q}$ is the operating firepower, $t_{\mathrm{test}}$ is the time duration of the test, $\eta_{\mathrm{th}}$ is the stove overall thermal efficiency, ${\bar{c}}_{p_{\mathrm{water}}}$ is the isobaric mass-specific approximate heat capacity of water between 20 °C and 100 °C given 4.180 kJ·kg ${}^{-1}$·K ${}^{-1}$ and $T_{0}$ is the temperature of the water at the beginning of the test.

Table 7 also presents convective heat-transfer parameters, namely the Reynolds number Re, the Nusselt number Nu and the convective heat-transfer coefficient *h*. However, Nu and *h* do not cover qualitative aspects of energy-transfer processes. As mentioned in Section 2.1.5, alternative assessment parameters are required to indicate how energy is used [39,41,42]. To achieve that, the stove system was divided into two energy-transfer unit components:

- The first component concerns adding thermal energy from combustion to flue gases. This unit operation is assessed by the energy-devaluation number noted $N_{h}$, introduced in Equation (30).
- The second component concerns transferring heat from flue gases to the pot. This unit is assessed by exergy destruction number $N^{E}$ in Equation (31).

Then, the energy utilization for the entire process is assessed by the overall exergy destruction number ${\hat{N}}^{E}$, see Equation (32). Table 8 presents a schematic of these alternative energy assessment parameters applied to the stove domain and calculation methods for the two consecutive energy-transfer components.

**Figure 8:**
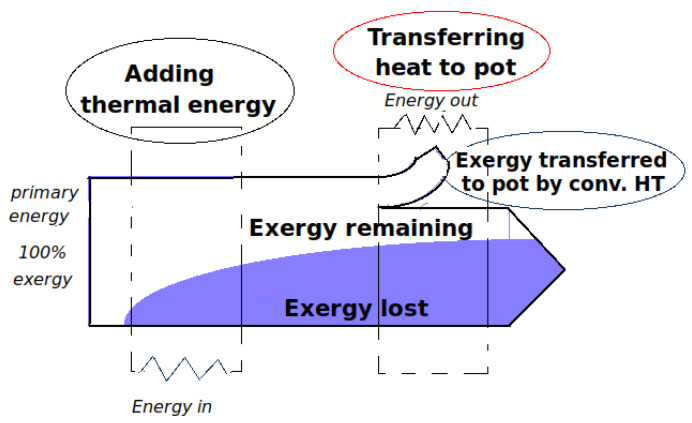
The value diagram of exergy destruction (loss) in a natural convection-driven woodburning stove operating with a pot.

Figure 8 represents a value diagram sketching the degradation of energy consecutive to heat-transfer processes through the stove.

Let us now show in practice, and step by step, the way to determine the alternative assessment numbers related to the devaluation chain of the two consecutive unit-transfer operations in the 4 in Elbow rocket stove of [15] taken as reference.

By writing an energy balance in this system, Table 9 presents, respectively, the rate of sensible enthalpy increase in the flue gases and the rate of exergy loss due to heat transfer. Then their difference gives the starting rate of exergy flow accompanied (in the last column) by the Carnot factor $\eta_{c_{1}}$ that assesses this flow work potential. To look closely, values of exergy flow rate in Table 9 are near to the rate of thermal energy transfer to pot ${\dot{Q}}_{c-p}$ proposed by [15] and included in Table 7.

It is worth noting that for a stove with a pot in place, difficulties may arise in terms of assuming a priori a proper temperature profile as the flame interacts with the pot surface. Therefore the exergetic Carnot factor of the heat transfer to the flue gases can be accessed indirectly:

(68) $$\eta_{c_{1}}=1-N_{h}=1-\frac{T_{0}\cdot {\dot{m}}_{A}\cdot \left(s\left(T_{\mathrm{exit}}\right)-s\left(T_{0}\right)\right)}{{\dot{m}}_{A}\cdot \left(h\left(T_{\mathrm{exit}}\right)-h\left(T_{0}\right)\right)}=1-\frac{T_{0}\cdot \left(s\left(T_{\mathrm{exit}}\right)-s\left(T_{0}\right)\right)}{\left(h\left(T_{\mathrm{exit}}\right)-h\left(T_{0}\right)\right)}$$

The Carnot factor $\eta_{c_{1}}$ is subjected to devaluation dictated by the overall exergy loss number ${\hat{N}}^{E}$, a concept largely developed in references like [39,44,48]. To evaluate ${\hat{N}}^{E}$, one needs to determine first the second exergetic Carnot factor $\eta_{c_{2}}$ for the subsequent convective heat transfer to pot. Thus, according to Equation (59), $\eta_{c_{2}}$ is given:

(69) $$\eta_{c_{2}}=\frac{T_{0}}{T_{p}}\left(1-\frac{T_{p}}{T_{g}}\right)$$

So following Equation (32), the overall exergy loss number ${\hat{N}}^{E}$ reads:

(70) $${\hat{N}}^{E}=\frac{T_{0}\cdot {\dot{m}}_{A}\cdot \left(s\left(T_{\mathrm{exit}}\right)-s\left(T_{0}\right)\right)}{\eta_{c_{2}}\cdot {\dot{Q}}_{c-p}}$$

This energy-transfer quality assessment number ${\hat{N}}^{E}$ can be interpreted as the ratio of the rate by which exergy in the flowing fluid is lost to the rate by which exergy is transferred from a convective heat-transfer process to the pot. Table 10 presents the resulting overall exergy loss number ${\hat{N}}^{E}$ for the 4 in Elbow woodburning cooking stove operating with pot.

Finally, the *global devaluated exergy factor* from the two consecutive unit operations is by definition:

(71) $$\eta_{E}=\eta_{c_{1}}\cdot {\hat{N}}^{E}$$

Table 11 summarizes numerical results of all qualitative assessment numbers for the two energy-transfer components of the 4 in Elbow cooking stove in application.

**Figure 9:**
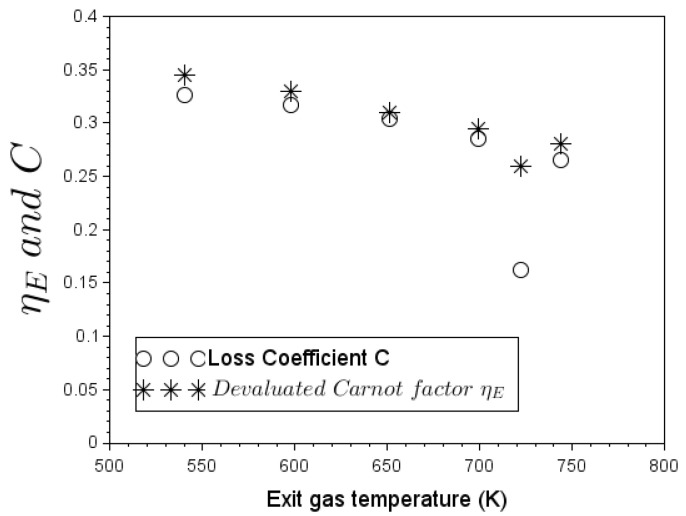
Values of devaluated exergy Carnot factor parameter compared to loss coefficient.

Figure 9 depicts values of the devaluated Carnot factor $\eta_{E}$ compared to the loss coefficient of the 4 in Elbow with pot. Note that raw data adopted for the adjustment of loss coefficient in Zube [15] have been collected from the experimental works of Agenbroad et al. [21,22,23].

A satisfactory agreement emerges between the devaluated exergy Carnot factor $\eta_{E}$ and the loss coefficient *C* at different exiting gas temperatures in Figure 9. The same decreasing trend of the loss coefficient when firepower increases in a woodburning stove equipped with a pot can be observed referring to experimental raw data in [23,46]. The form of the overall exergy destruction number ${\hat{N}}^{E}$ in Equation (70) becomes very instructive in terms of explaining the decline of the overall exergy Carnot factor or the stove loss coefficient.

To take values in Table 11 as an example, the overall exergy destruction number ${\hat{N}}^{E}$ for the first test is roughly equal to unity. This means that the rate by which exergy is being transferred to the pot is nearly the same as the rate by which exergy would be lost within the flowing fluid. However, for the rest of the tests, as firepower increases, the drop of ${\hat{N}}^{E}$ means that the *entropic potential* of convective heat transfer becomes much higher than that generated in the working flow; therefore, the availability to set fluid in motion decreases. This concept of entropic potential in energy-transfer operations is largely developed in [48].

Futhermore, it appears that exergy destruction effects become important when convective heat-transfer potential increases. The devaluated exergy factor for the overall process $\eta_{E}$ in Equation (71) is indeed a product of two competing terms: $\eta_{c_{1}}$ and ${\hat{N}}^{E}$. When flue gas temperature increases, the Carnot factor $\eta_{c_{1}}$ increases as well, but in contrast the overall exergy destruction number ${\hat{N}}^{E}$ decreases.

## 5. Conclusions

The second law of thermodynamics analysis was performed to assess the loss coefficient in buoyantly-driven biomass cooking stoves. Accordingly, a simplified mathematical model of the entropy-generation rate in the flow field was developed. To validate the model, experiments were conducted first on a G3300 woodburning cookstove operating without pot to better isolate physical processes governing the basic behaviour of the stove. For the practical case of a stove operating with the cooking pot in place, data from published literature have served for validation. In particular, mass-flow rate and flue gas temperature at different firepower levels have been monitored.

For the parameters under study, it turned out that the entropy generation due to fluid friction is negligible compared to the global dissipation process. Therefore, heat-transfer processes are revealed to be the main source of irreversibilities in the flowing fluid. Energy flux applied as flow work in cooking stove is pure exergy which is lost in consecutive dissipative processes. Furthermore, analysis shows:

- In the stove without pot: Experimental values of the stove loss coefficient at different exhaust-gas temperatures coincide with the heat Carnot factor. Thus, the energy transfer in the cookstove becomes thermodynamically assimilable to a reversible engine that releases its work output into buoyant flow work. This thesis leads to a novel definition of the loss coefficient as a measure of exergy flow.
- In the stove with a cooking piece (pot) in place: As upward hot gases transfer exergy to pot, both the transferred thermal energy and the needed flow work degrade. Alternative heat-transfer parameters such as exergy Carnot factor and energy-devaluation numbers were introduced to account for the destruction of exergy in the overall process. A clear relationship emerged between devaluated exergy Carnot factor and experimental values of the loss coefficient at different flue gas temperatures.

The second law analysis somewhat changes the paradigm in stove engineering by bringing quite a different perspective to the traditional concept of the so-called *loss coefficient*. From now, this *flow loss coefficient* can rather be regarded as the *availability* of internal energy to generate (buoyant) flow work through the stove. Therefore, the magnitude of this *reversible work* depends upon operating conditions and consecutive energy-transfer processes undergone following the stove operating at high or low firepower levels. Minimizing entropy generation with a view to optimizing energy-transfer processes in biomass cooking stoves remains a potential application for future works.

## Figures and Tables

**Table 1:** Loss coefficient *C* in small-scale biomass cooking stove modelling.

Reference Authors	System Configuration	Highlights of the Study
MacCarthy [9]	Open cooking fire,	The study referred to various correlations in literature.
	Shielded cooking	Fluid flow constants and equations have been collected,
	fire.	deduced from a general balance of forces.
		However any specific value of *C* has been reported.
Agenbroad	Stove without pot	Analytical stove flow modelling considered by default
[21,22,23]	and Stove with pot.	*C* remaining constant for stove operations:
		(a) *C*= 0.5 for stove without pot.
		(b) *C* = 0.35 for stove with pot.
		However, variable *C* depending on operating firepower
		level was experimentally observed.
		In theory, model accounted contributions for both
		losses due to viscous effects and losses due to
		distributed heat addition. In the assumption of a more
		realistic linear density profile, model suggested to
		replace *C* by the product $C=C_{viscous}\cdot C_{heat}$. Reduction
		of available chimney effect results in $C_{heat}=\frac{\sqrt{2}}{2}\approx 0.707$.
		CFD-based loss coefficient predicted stove behaviour
		using pressure drop with comparison to validation
		results [21].
		Effects for reacting flow are unknown.
Kshirsagar [24]	Stove with pot.	Model treated *C* as a variable which in itself depends
		upon other variables, i.e., inlet area and geometrical
		variation.
		Model predicted *C* in the range of 0.195–0.38.
		Effects for reacting flow are unknown.
Zube [15]	Stove with pot.	Model adapted for calculation experimental *C* values
		formerly determined in [21,23]. Model discussed
		heat-transfer efficiency of the three different HT modes.
		Theoretical calculations in MathCAD/Excel established
		some correlations between *C*, firepower, convection and
		combustion efficiency, pot gap adjustment, pot skirt
		adjustment, skirt height, etc.
Parajuli [25]	Two-pot enclosed	Mass-flow rate calculated on the pressure difference
	mud cookstoves.	incorporating geometric loss coefficients to determine *C*.
		Thermal effects are not taken into account.

**Table 2:** G3300 stove model geometrical parameters.

Parameter	Value	Unit
Chimney diameter	100	mm
Chimney height	220	mm
Outer stove diameter	230	mm
Air entrance area	160 × 100	mm${}^{2}$
Entrance area/Chimney area ratio	2.04	–

**Table 3:** Elemental analysis of Sapele.

Element	Percentage (%)
Carbon	54.6
Hydrogen	4.7
Oxygen	40.7
Sulfur	0
Nitrogen	0.01

**Table 4:** Fuel batch loads.

# test	Stack Wood	Entrance Area (m${}^{2}$)	*IAR*	Fuel Burning Rate (kg·s${}^{-1}$)
1	$\frac{1}{2}$ +$\frac{1}{2}$ sticks	0.0153	0.95625	0.027 × 10${}^{-3}$
2	2 sticks	0.01475	0.921875	0.037
3	1 +$\frac{1}{2}$ sticks	0.015	0.9375	0.050
4	1 +$\frac{1}{2}$ sticks	0.015	0.9375	0.059
5	$\frac{1}{2}+\frac{1}{2}$ sticks	0.0153	0.95625	0.085
6	$\frac{1}{2}+\frac{1}{2}+\frac{1}{2}$ sticks	0.015	0.9375	0.128
7	2 sticks bis	0.01475	0.921875	0.149
8	2 sticks	0.01475	0.921875	0.170
9	4 sticks	0.0135	0.84375	0.219
10	3 sticks	0.014	0.875	0.229
11	4 sticks	0.0135	0.84375	0.309
12	2 +$\frac{1}{2}$ sticks	0.0144	0.9	0.314

**Table 5:** Fuel burning rate, Firepower, Bulk flow temperature, Air mass-flow rate and entropy-generation rate for a G3300 woodburning stove without cooking pot.

		Experimental Results				Table Values	
#Test	${\dot{m}}_{F}$	Firepower	$T_{H}$	$O_{2}$	${\dot{m}}_{A}$	$s\left(T_{H}\right)-s\left(T_{0}\right)$	${\dot{S}}_{{\mathrm{gen}}_{1}}$
	**(kg**·**s**${}^{-1}$)	**(kW)**	**(K)**	**(%)**´	**(kg**·**s**${}^{-1}$**)**	**(kJ**·**kg**${}^{-1}$·**K**${}^{-1}$)	**(kJ**·**K**${}^{-1}$·**s**${}^{-1}$**)**
1	0.027 × 10${}^{-3}$	0.5	421	19.5	1.08 × 10${}^{-3}$	0.346	0.37 × 10${}^{-3}$
2	0.034	0.63	439	19.35	1.26	0.353	0.44
3	0.050	0.94	527	18.78	0.60	0.558	0.89
4	0.059	1.1	543	18.54	2.33	0.608	1.42
5	0.085	1.6	484	17.85	2.21	0.460	1.02
6	0.128	2.4	684	16.36	2.58	0.871	2.25
7	0.149	2.8	694	17.53	3.64	0.888	3.23
8	0.170	3.2	800	17.43	4.08	1.059	4.32
9	0.219	4.1	818	14.66	3.55	1.084	3.85
10	0.229	4.3	873	14.66	3.52	1.158	4.08
11	0.309	5.8	995	11.38	3.75	1.292	4.84
12	0.314	5.9	994	12.64	4.21	1.291	5.44

**Table 6:** The rate of exergy by heat transfer and associated parameters for the G3300 stove operating without cooking pot.

Firepower	$T_{H}$	${\dot{m}}_{A}$	Δθ	${\dot{X}}_{{\mathrm{destr}}_{\left(\mathrm{heat}\right)}}$	$\Delta {\dot{X}}_{\mathrm{flow}}$	$\eta_{c_{1}}$
(kW)	(K)	(kg·s${}^{-1}$)	(kW)	(kW)	(kW)	
0.5	421	1.08 × 10${}^{-3}$	0.133	0.111	0.022	0.16
0.63	439	1.26	0.179	0.133	0.032	0.18
0.94	527	1.60	0.372	0.266	0.096	0.26
1.1	543	2.33	0.598	0.422	0.165	0.28
1.6	484	2.21	0.416	0.303	0.093	0.22
2.4	684	2.58	1.029	0.669	0.369	0.36
2.8	694	3.64	1.490	0.964	0.542	0.36
3.2	800	4.08	2.140	1.287	0.885	0.41
4.1	818	3.55	1.932	1.147	0.814	0.42
4.3	873	3.52	2.130	1.215	0.943	0.44
5.8	995	3.75	2.783	1.443	1.349	0.48
5.9	994	4.21	3.120	1.619	1.511	0.48

**Table 7:** Mass-Flow Rate, Temperature and Their Effects on Heat Transfer—4 in Elbow with pot at different operating firepower.

FP	${\dot{m}}_{A}$	$T_{g}$ (at Pot Bottom)	^1^ $T_{\mathrm{exit}}$	*V*	Re	Nu	*h*	${\dot{Q}}_{c-p}$
(kW)	(kg·s${}^{-1}$)	(K)	(K)	(m·s${}^{-1}$)			(kW·m${}^{-2}$·K${}^{-1}$)	(kW)
1.5	3.08 × 10${}^{-3}$	619	540	0.72	1352	17.2	8.3 × 10${}^{-3}$	0.245
2	3.00	705	598	0.78	1176	16.2	8.4	0.322
2.5	2.87	792	651	0.83	1027	15.0	8.6	0.405
3	2.67	879	698	0.88	911	14.2	8.6	0.482
3.5	2.45	965	744	0.85	752	12.9	8.4	0.542
4	1.51	1052	722	0.57	438	9.8	8.8	0.499

^1^ Adapted from Zube [15].

**Table 8:** Energy-devaluation number $N_{h}$, Exergy destruction number $N^{E}$, Overall exergy destruction number ${\hat{N}}^{E}$ and Entropy indirect calculation methods for the two consecutive energy-transfer components of the cooking stove sketched in Figure 8.

	Energy Transfer Component	${\dot{S}}_{\mathrm{gen},i}$	$N_{i}$
$N_{h}$	adding thermal energy		
energy devaluation	to flue gases	${\dot{m}}_{A}\cdot \left(s\left(T_{\mathrm{exit}}\right)-s\left(T_{0}\right)\right)$	$\frac{T_{0}\cdot {\dot{m}}_{A}\cdot \left(s\left(T_{\mathrm{exit}}\right)-s\left(T_{0}\right)\right)}{{\dot{m}}_{A}\cdot \left(h\left(T_{\mathrm{exit}}\right)-h\left(T_{0}\right)\right)}$
number			
$N^{E}$	transferring heat		
exergy destruction	from flue to pot	${\dot{m}}_{A}\cdot \left(s\left(T_{\mathrm{exit}}\right)-s\left(T_{g}\right)\right)$	$\frac{T_{0}\cdot {\dot{m}}_{A}\cdot \left(s\left(T_{\mathrm{exit}}\right)-s\left(T_{g}\right)\right)}{\dot{E}}$
number	(conv. heat transf)		
${\hat{N}}^{E}$			
**Overall exergy**	**transferring exergy**	${\dot{m}}_{A}\cdot \left(s\left(T_{\mathrm{exit}}\right)-s\left(T_{g}\right)\right)$	$\frac{T_{0}\cdot {\dot{m}}_{A}\cdot \left(s\left(T_{\mathrm{exit}}\right)-s\left(T_{g}\right)\right)}{\eta_{c_{2}}\cdot {\dot{Q}}_{c-p}}$
**loss number**	**to the process**		

**Table 9:** Sensible enthalpy increase, loss of exergy due to heat transfer to flue gases, exergy flow due to convective heat transfer to pot for a 4 in Elbow with pot at different operating firepowers.

		Sensible Enthalpy	Loss of Exergy Due	Exergy Flow Consecutive	$\eta_{c_{1}}$
Firepower	$T_{\mathrm{exit}}$	Gained by Flue Gases	to Heat Transfer to Flue Gases	to Heat Addition	
		${\dot{m}}_{A}\cdot \left(h\left(T_{\mathrm{exit}}\right)-h\left(T_{0}\right)\right)$	$T_{0}\cdot {\dot{m}}_{A}\cdot \left(s\left(T_{\mathrm{exit}}\right)-s\left(T_{0}\right)\right)$	${\dot{X}}_{\mathrm{heat}}$	
**(kW)**	**(K)**	**(kW)**	**(kW)**	**(kW)**	
1.5	540	0.780	0.530	0.250	0.32
2	598	0.944	0.606	0.338	0.36
2.5	651	1.065	0.650	0.415	0.39
3	698	1.127	0.660	0.467	0.42
3.5	744	1.154	0.650	0.504	0.44
4	722	0.675	0.387	0.288	0.43

**Table 10:** Exit gas temperature, Exergy losses along the bottom surface of the pot to the exit after impinging, Exergy transferred to pot and Overall exergy destruction number ${\hat{N}}^{E}$ in a 4 in Elbow with pot at different operating firepower levels.

Firepower	$T_{\mathrm{exit}}$	$T_{0}\cdot {\dot{m}}_{A}\times \left(s\left(T_{\mathrm{exit}}\right)-s\left(T_{g}\right)\right)$	$\eta_{c_{2}}\cdot {\dot{Q}}_{c-p}$	${\hat{N}}^{E}$
(kW)	(K)	(kW)	(kW)	
1.5	540	0.123	0.117	1.05
2	598	0.144	0.156	0.92
2.5	651	0.163	0.205	0.80
3	698	0.177	0.251	0.71
3.5	744	0.185	0.286	0.65
4	722	0.165	0.266	0.62

**Table 11:** Exergetic Carnot factors and Energy-devaluation numbers in a 4 in Elbow with pot at different operating firepowers and exit-gas temperatures.

Firepower	$T_{\mathrm{exit}}$	$N_{h}$	$\eta_{c_{1}}$	$\eta_{c_{2}}$	${\hat{N}}^{E}$	$\eta_{E}$
(kW)	(K)					
1.5	540	0.68	0.32	0.48	1.05	0.34
2	598	0.64	0.36	0.49	0.92	0.33
2.5	651	0.61	0.39	0.51	0.80	0.31
3	698	0.58	0.42	0.52	0.71	0.30
3.5	744	0.56	0.44	0.53	0.65	0.29
4	722	0.57	0.43	0.53	0.62	0.27

## Data Availability

Not applicable.

## Abbreviations

The following abbreviations are used in this manuscript:

MDPI	Multidisciplinary Digital Publishing Institute
DOAJ	Directory of open access journals
TLA	Three letter acronym
LD	Linear dichroism
